# Transactions of the Chicago Society of Physicians and Surgeons, September 28, 1874

**Published:** 1874-10-15

**Authors:** Ralph E. Starkweather


### TRANSACTIONS OF THE CHICAGO SOCIETY OF PHYSICIANS AND SURGEONS.

REGULAR MEETING, SEPTEMBER 28, 1874.

*Reported by Ralph E. Starkweather, M.D.*

DR. J. E. OWENS, Vice-President, in the chair. Dr. Owens gave briefly a report of two cases of fracture treated at St. Luke's Hospital, in one of which, that of a colored man thirty-eight years of age, a piece of bone the size of a filbert had been broken off from the left fibula, about two inches above the external malleolus. The case progressed favorably under the usual treatment (by Dupuytren's Splint), the fragment of bone speedily united and became incorporated with the long bone itself. The second case, a fracture of the humerus, just above the condyles, was of interest from the fact that it was caused by the throwing of a base ball.

Dr. H. A. Johnson addressed the society upon the subject of pneumatic aspiration, illustrating the same by exhibiting and explaining the several forms of instruments now employed, exposing at the same time defects, both in theory and construction, of some of the aspirators. The following is a synopsis of his lecture:

In the first place the question arises as to what is understood by the term aspiration. Is it anything new either in principle or mechanism, or is it merely after the fashion of the stomach pump? Some physicians deny that there is any new principle involved in pneumatic aspiration. In my judgment, Dieulafoy has introduced a new idea and instrument. It consists in this: the previous production of a vacuum, and then the connection of it with a cavity containing fluid. The second question that presents itself, is that of the methods of producing this vacuum. The aspirator of Dieulafoy was then explained. It consists of a glass cylinder, in which there is a piston, moved by means of a rack and pinion. The aspirator is inclosed in a metal frame, having a broad and heavy base. There is attached a graduated scale to measure fluids, and also two stop-cocks. The canules and needles and rubber tubing were also exhibited.

Dr. Johnson showed another form of aspirator, known as the *Aspirateur Potain*, to which, for reasons hereafter to be given, he gave the preference. In referring to an instrument made in Boston, a modification of Potain's, he said that it would be unnecessary to purchase the bottle accompanying it, as the perforated rubber cork, with its double canula, would answer every purpose, and bottles were always easily to be found in most families.

The next step is to place the vacuum in relation with the cavity to be emptied. As regards the choice of a needle and canula, there are two kinds of needles to use, each suited to its particular purposes. One of the needles is like the ordinary one used in hypodermic injections, but this is objectionable in cases where the sharp penetrating point going beyond the wall of the cavity, might injure delicate viscera, as for example the lung.

In many respects the needles and canules, which differ from the needles first described, of the Aspirateur Potain, are greatly to be preferred. This, the second variety, is really a trocar with a canula, with a branch tube for the attachment of the soft rubber connection. In case of obstruction the canula can be cleared with a celerity and ease quite impossible in the first variety of needle.

The method of puncture was next explained, and that it was necessary first to determine where you want to put the point, and then how deeply the puncture should be made.

In some cases there are gases as well as fluids in a cavity, and by this means the vacuum in some forms of aspirators may be disturbed if not destroyed.

The application of pneumatic aspiration has been widely discussed, of late years, in the medical journals. During the past two years, in which Dr. Johnson has employed this process, and in upwards of seventy different cases, he has never seen, in a single instance, any particular discomfort to the patient, from its use. Among the numerous cases reported, the particulars of a case of an obscure and deep-seated pain in the mammary region, which, upon exploratory puncture an inch and one-quarter deep, by the aspirator, proved to be an abscess, were most instructive. In another case, that of acute infantile hydrocephalus, he had introduced the needle of the aspirator into the anterior fontanelle, to the right of and towards the median line, the point directed obliquely, so as to penetrate the membranes beneath the vessels of the longitudinal sinus. A little fluid was drawn off, with temporary relief to the breathing and circulation. There were no unfavorable symptoms attributable to the operation. The patient, however, subsequently died. In another case, where there was acute effusion in the knee joint, pneumatic aspiration afforded prompt and decided relief; but in forty-eight hours the effusion had returned.

The great assistance to be derived from pneumatic aspiration in differential diagnosis was illustrated by the case of a patient who had been under the care of a homœopathist for so-called uterine trouble, with reflex uterine irritation. Upon examination, it was found that the apex of the heart was under the axilla, and that the right chest measured two inches larger than the left. The question then arose as to whether there was a solid morbid growth in the thorax, or fluid only. Upon introducing the canule of the aspirator into the chest, upwards of two and one-half quarts of muddy serum, not yet quite purulent, was drawn off. The patient for the past year, since that event, has been in perfect health.

The subject of injury to the lungs by aspiration by a penetrating wound of the needle, was then discussed.

Several cases of great interest and value were cited, going to show that the danger to the lung in any event would be little or none. A noticeable feature in pneumatic aspiration, is that by this operation there is little probability of introducing from without inwards any points for future infection of the system. Preference was given to the Aspirateur Potain over that of Dieulafoy, because the vacuum was less uncertain, and is more easy to be maintained. It was not considered to be a point of advantage in Dieulafoy's aspirator, that it might be used for the injection into cavities of medicated fluids, inasmuch as it would be like using an unwashed syringe lately containing unhealthful fluids (pus, for example), to throw new and medicated fluids into the body.

Dr. Owens reported a case under the care of Dr. Heydock, in which pneumatic aspiration had been tried upon an effusion in the pericardial sac of a patient who had pericarditis and endocarditis.

At the time of the operation, the pulse was 126, temperature $101\frac{3}{4}$° F. and respiration 66. The precordial dullness extended from the first rib to the upper margin of the sixth; apex impulse indistinctly felt in the fifth intercostal space. Laterally the percussion dullness began at about the middle line of the sternum, and extended around to the left side. The needle of the aspirator was inserted in the fourth intercostal space, one inch and a half from the left margin of the sternum. It was pushed upwards, inwards, and a little backwards. Frothy, dark-colored blood appeared in the receiving bottle, probably because the lung had been pierced. Three-quarters of an hour after this operation, the pulse was 120 and the respiration 45. Dieulafoy remarks of *adults* that when the needle has punctured from 1.17 to 2.34 inches, the instrument must meet either the heart or the effusion. The head and shoulders should be elevated with pillows during the operation, and the needle plunged in at the close of an expiraation. This patient, a boy fifteen and a half years old, afterwards died. The autopsy revealed perfect agglutination of the pericardial layers.

Dr. Johnson gave an account of a similar operation he had performed upon a patient at the county hospital, and of experiments he had made upon the cadaver. The puncture should be two and a half inches from the left border of the sternum; if you go nearer to the sternum you must have a longer needle; there were some reasons which inclined him to think favorably of puncturing in the fifth intercostal space, but as his experiments had not yet been concluded, he reserved his opinion upon this point.

Dr. Emmons read a report of a case of uterine interstitial fibroid tumor, treated hypodermically by ergotine, of which the following is an abstract:

Mrs. X, married, age 45, American, the mother of three children, the youngest of whom is eleven years ot age, was in good health till the time of her last parturition, which proved to be a difficult one, followed by hourglass contractions, and permanent impairment of health. Two years ago a noticeable change had gradually taken place: menstruation had become copious, and more prolonged than usual, requiring patient to keep the bed for ten days at a time. She was emaciated, had sciatica and chronic laryngitis. At the time of Dr. Emmons' first visit, December, 1873, in addition to above symptoms, the patient had become so exsanguinous as almost to be in a state of syncope, from constant flooding; there was pain in the uterus and chest; loss of appetite and sleep. There was abdominal tenderness, particularly over the region of the right ovary. The uterus was enlarged, extending up to within two inches of the umbilicus, and a little higher upon the right than left side.

Digital examination, per vaginam, revealed anteversion, the os low down against rectum, and dilated to about one-half inch. A metallic sound could be introduced (with strong curve towards anterior wall of abdomen) to a depth of six and one-half inches; a flexible catheter passed one-half inch farther. There was a hard tumor commencing at middle of the neck of the uterus, attached to its anterior wall, and extending to the fundus uteri.

Treatment: directed a tonic of iron and quinine, and

℞.—Ergotine (Bonjeau) ...... ʒ j.

Aqua distil............ f. ℥ j.

Glycerini ............ f. ʒ j.

M. Twelve drops, daily, hypodermically.

Improvement was immediate, as shown by the subsidence of pain and hæmorrhage. At the end of fifty-nine days the patient was much better, pulse fuller, appetite better, sleeps well and without anodynes, and able to sit up two hours daily; less abdominal tenderness, but no diminution in size of tumor. The dose of ergotine was increased so as get about four minims per dose, and continued for three weeks, without any particular change in patient's condition.

The use of Squibb's aqueous solution of ergot, prepared for me so as to get one grain of the drug to the minim, giving twenty minims daily, by hypodermic injection over the deltoid, was most satisfactory.

Examination twelve days after this change of medicine, revealed the tumor subsided to four and one-half inches below umbilicus. The cavity of uterus measured about five and one-half inches in depth. Patient able to ride out daily; has gained eighteen pounds during the past fortnight; is entirely free from pain; last menstruation normal in quantity, and lasted four days. At another examination in June the fibroid had still farther diminished in size, the uterine cavity measured four inches in depth. Patient complained at this time of a pretty free watery discharge from the vagina. A month later, during which time the treatment had been continued, the uterine cavity was found to measure three and one-half inches in depth, watery discharge still present. The treatment was suspended for ten days and then resumed. In August the uterus had regained its normal size, and no tumor remained. The continued daily hypodermic injection, during the period of 156 days, of the twenty minims of a preparation containing Squibb's aqueous solution ot ergot, had the effect of gradually reducing the fibroid, till hardly a trace of it could be found. There was no particular inconvenience or irritation produced at the points of puncture, especially after laying aside the use of the alcoholic preparation of ergotine.

Dr. Emmons gave some quotations regarding the action of ergot, from the results of an experimental investigation by Dr. Warnich, and from recent works of other authorities, such as Drs. Paul Vogt, Ritchie, Handelin, and C. Boldt.

*In true interstitial fibroid of the uterus, treated hypodermically with the aqueous solution of ergot, we may expect in the large majority of cases eminently more satisfactory results than by any other mode of treatment, or by operation.*

Dr. Davis.—Why was the medicine not administered by the stomach ?

Dr. Emmons replied, because the effect was more satisfactory when given hypodermically; it enters more rapidly into the circulation, and does not disturb the appetite or digestion. He knew of a physician who had given the ordinary tincture of ergot by injection, and thereby quickly produced numerous abscesses.

Dr. Merriman said that in a couple of cases under his care there have a quantity of abscesses followed upon the hypodermic use of a solution of ergotine. Afterwards he gave Squibb's extract of ergot, in solution, by the mouth, but it was not so efficient as when given by injection. He had always made the insertion directly over the region of the uterus. Even in treating cases not of the interstitial form of fibroids, when giving ergot by the mouth, great benefit has been apparent; the debility is lessened, the menstruation has become more regular in amount and time, the patient feels better, even when there has been no diminution in the size of uterine growth.

The society next discussed the report of the committee on the medical fee bill, but, owing to the lateness of the hour, adjourned, leaving the question of its adoption to a future meeting.

